# Redo aortic valve replacement due to early structural valve deterioration in a trifecta valve: A case report

**DOI:** 10.1016/j.ijscr.2021.106381

**Published:** 2021-09-06

**Authors:** Tatsuya Watanabe, Noriyuki Tokunaga, Keita Maruno, Hideo Yoshida, Masahiko Kuinose

**Affiliations:** Department of General Surgery, Kawasaki Medical School General Medical Center, 2-6-1, Nakasange, Kita-ku, Okayama 700-8505, Japan

**Keywords:** Trifecta, Structural valve deterioration, Aortic regurgitation, Case report

## Abstract

**Introduction and importance:**

While the number of SAVR cases has been increasing for patients below their sixties due to the improvement of bioprosthetic valves, some early structural valve deterioration (SVD) in Trifecta valves has been reported.

**Case presentation:**

We present a case of a female who presented with sudden shortness of breath. Ultrasonography diagnosed SVD. We performed redo aortic valve replacement due to SVD in Trifecta valve. With our surgical technique we could remove the bioprosthetic valve easily.

**Clinical discussion:**

We could easily remove the mounted prosthetic valve along with the titanium band. These cases may emerge with acute heart failure due to sudden massive aortic regurgitation, not like the gradual progression of stenosis due to calcification.

**Conclusion:**

The postoperative course in Trifecta recipients must be followed carefully.

## Highlights

• Early structural valve deterioration with Trifecta valve
• Postoperative patients using Trifecta valve should be carefully followed up.
• Mounted Trifecta valve could easily be removed along with titanium band.

## 1 Introduction and importance

The number of transcatheter aortic valve replacement (TAVR) cases has been increasing recently for the treatment of aortic valve stenosis (AS) [1], [2], [3], [4]. However, because the long term outcomes of TAVR are not yet known, the Japanese guidelines for valvular disease (JCS/JATS/JSVS/JSCS 2020 Guidelines on the Management of Valvular Heart Disease) recommend surgical aortic valve replacement (SAVR) for patients under 75 years-old. Also, 2021 ESC/EACTS Guidelines recommend SAVR for patients under 75 years-old if they are low-risk for SAVR [5]. 2020 ACC/AHA Guidelines recommend SAVR for patients under 65 years of age or with life expectancy more than 20 years [6].

While the number of SAVR cases has been increasing for patients in their sixties due to the improvement of bioprosthetic valves, some early structural valve deterioration (SVD) in Trifecta valves has been reported [7], [8], [9]. Here, we report on our experience with a reoperation for early SVD in a Trifecta GT valve four years after the initial SAVR operation as well as our technique to remove the implanted valve. Our work has been reported in line with the SCARE 2020 criteria [10].

## 2 Case presentation

A 71-year-old woman presented with sudden shortness of breath. Four years prior, she had a SAVR using a Trifecta GT aortic valve for bicuspid aortic valve stenosis. Her initial postoperative course was stable, but four years after the valve implant, she had symptoms of acute heart failure and was taken to another hospital. There, she was diagnosed with acute heart failure due to SVD in the Trifecta valve and was then referred to our hospital for SAVR reoperation after the management of her heart failure. Her preoperative heart failure was stable with a few diuretics and beta-blocker. There was no specific medical history other than her heart failure.

**Fig. 1:**
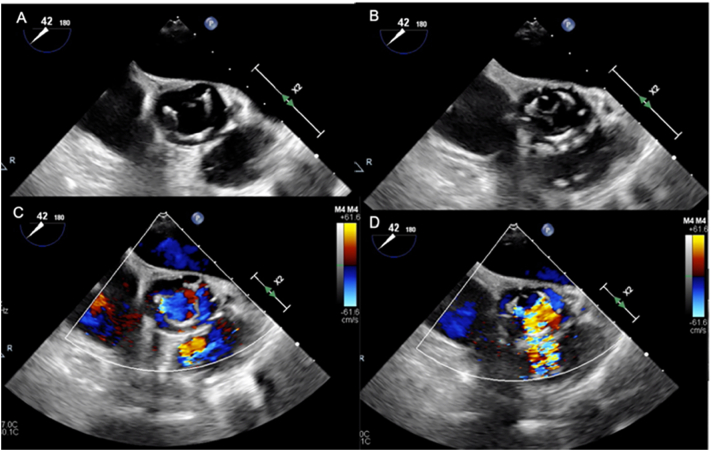
Transesophageal echocardiography. Short axis view; A: systolic phase, B: diastolic phase. Short axis view with color doppler; C: systolic phase, D: diastolic phase.

**Fig. 2:**
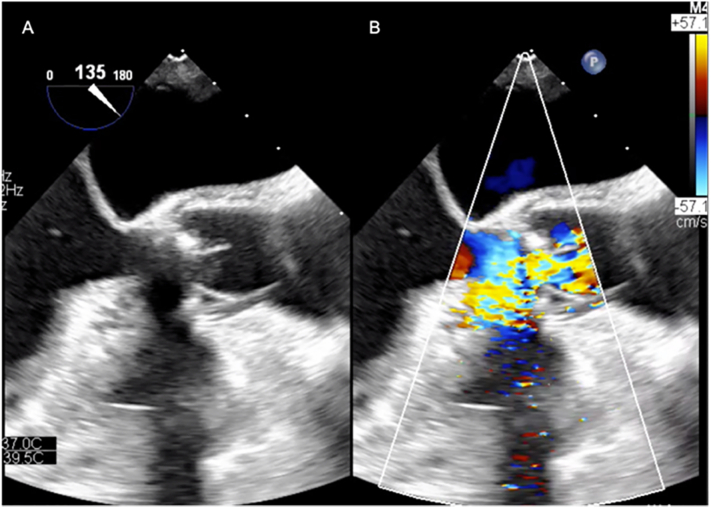
Transesophageal echocardiography. Long axis view; A: diastolic phase, B: diastolic phase with color doppler.

Preoperative blood tests showed the following results: hemoglobin, 9.5 g/dL; WBC, 3.70 10^3^/μL; C-reactive protein, 0.09 mg/dL; creatinine, 1.06 mg/dL; urea nitrogen, 24 mg/dL; platelet, 172 10^3^/μL. Electrocardiography showed a first-degree atrioventricular (AV) block without any ST-T changes. A preoperative transthoracic echocardiography (TTE) showed normal left ventricular systolic function with a left ventricular diameter of 55 mm (diastolic) and 38 mm (systolic), a left ventricular ejection fraction of 62%, a stroke volume of 77.7 mL, an E/A ratio of 1.8, a 13.3 e/e’ ratio, and a deceleration time of 188 ms. The left ventricular end-diastolic volume was 121 mL, and the end-systolic volume was 46 mL. The TTE also showed severe trans-valvular aortic valve regurgitation, abdominal aortic regurgitant wave and moderate MR. Intraoperative esophageal echocardiography showed massive regurgitation between the N-L commissure and the left prosthetic leaflet (Fig. 1, Fig. 2).

**Fig. 3:**
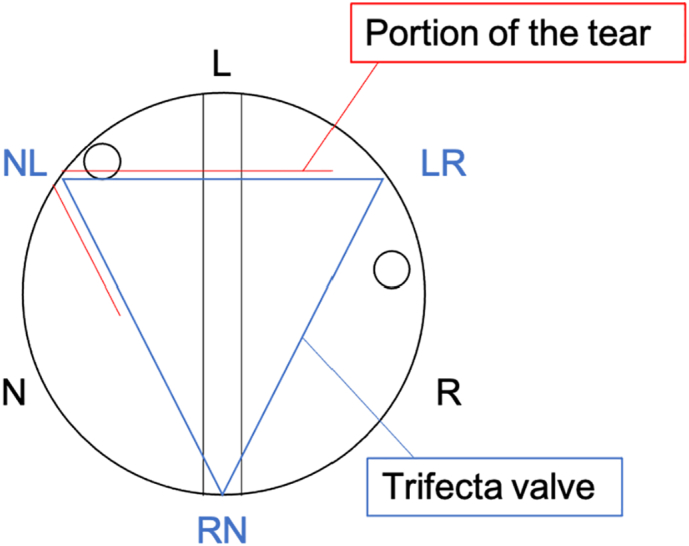
A Schematic diagram of the position between coronary ostium and Trifecta valve.

**Fig. 4:**
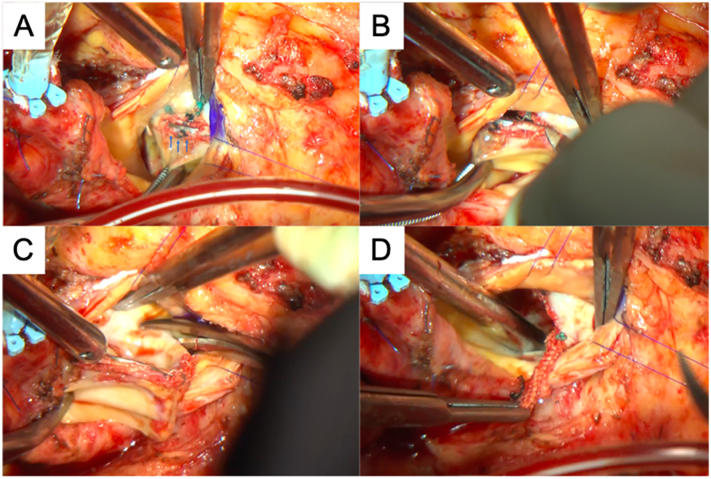
A: The titanium band was shown by cutting the suturing cuff. B: Cutting suturing cuff along with the titanium band by scalpel. C: After cutting with scalpel, easily resected with scissors. D: Removing the fabric part of the cuff from the annulus.

**Fig. 5:**
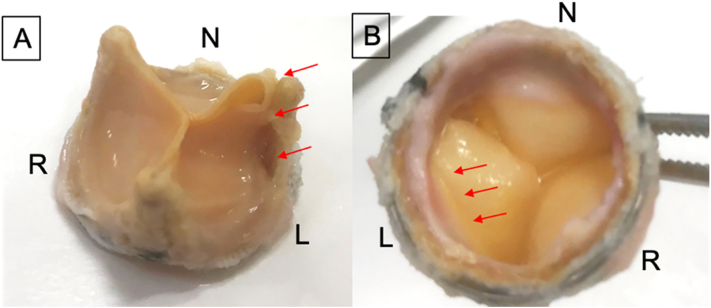
Removed Trifecta valve. A: teared cusp around N-L stentpost, B: pannus formation around the cuff.

Main operator was the professor of our institution, which is a university medical center. We approached with a median sternotomy and a cardiopulmonary bypass was established to the ascending aorta with bicaval drainage. The ascending aorta was clamped and antegrade cardioplegia was administered. Then the ascending aorta was opened and additional cardioplegia was selectively administered. Coronary arteries were placed on the opposite side due to her type 0 bicuspid valve. Fig. 3 is a schematic diagram which shows the position relationship [11]. The leaflet was torn between the N-L commissure stent and the left cusp of the Trifecta GT valve. With gentle traction on the L-R stent, we cut the cuff along the stent with a scalpel until we found the titanium band (Fig. 4A). We cut the cuff along with the titanium band while leaving the fabric part of the cuff at the annulus (Fig. 4B, C). After removing the main part of the valve, we then resected the fabric part of the cuff from the annulus (Fig. 4D). After removing the cuff, an Inspiris Resilia aortic valve (Edwards lifesciences, California, US) was placed at the supra-annular position. Fig. 5 showed the removed Trifecta valve which showed that the leaflet was torn between the N-L commissure stent and the left cusp, it also showed the pannus formation around the cuff and cusps.

Postoperative TTE showed normal left ventricle function with a left ventricular diameter of 41 mm (diastolic) and 28 mm (systolic), a left ventricular ejection fraction of 57%, a stroke volume of 45.9 mL, a 16.3 e/e’ ratio, and a deceleration time of 150 ms. The left ventricular end-diastolic volume was 63 mL and the end-systolic volume was 27 mL. There was no valve leakage. The postoperative course was stable.

## 3 Clinical discussion

The hemodynamic advantage of the Trifecta valve is reportedly due to the valve leaflets being mounted on the outside the sewing ring [12], [13]. However, in the past few years, there have been a number of reports of Trifecta valve leaflet tears leading to early SVD [7], [8], [9].

A re-operative SAVR should be considered when bio-prosthetic SVD happens in younger patients, such as in our case. It is reported that removing the mounted prosthetic valve is the main cause of extended operation and cardio-pulmonary bypass times [14].

As we described the Trifecta valve can be easily removed by cutting the cuff along with the titanium band. There are many types of prosthetic valves which have a metal band in their structure that this method can be applied to.

Most publications about Trifecta early SVD report tears at or around the stent post. These cases may emerge with acute heart failure due to sudden massive aortic regurgitation, not like the gradual progression of stenosis due to calcification. Though all tissue prosthetic valves need to be surveyed to identify features of deterioration, the postoperative course in Trifecta recipients must be followed carefully.

## 4 Conclusion

We experienced a redo surgical case of early SVD in a Trifecta valve four years after a SAVR for a type 0 bicuspid aortic stenosis. We could easily remove the mounted prosthetic valve along with the titanium band. The postoperative course in Trifecta recipients must be followed carefully.

## Sources of funding

None.

## Ethical approval

N/a

## Consent

Written informed consent was obtained from the patient for publication of this case report and accompanying images. A copy of the written consent is available for review by the Editor-in-Chief of this journal on request.

## Author contribution

TW, NT and MK performed surgery. TW and KM drafted the manuscript. HY and MK revised the manuscript critically for important intellectual content.

## Guarantor

Tatsuya Watanabe is the guarantor of this case report.

## Provenance and peer review

Not commissioned, externally peer-reviewed.

## Declaration of competing interest

The authors declare that they have no conflicts of interest concerning this article.
